# Using intermittent pulse oximetry to guide neonatal oxygen therapy in a low-resource context

**DOI:** 10.1136/archdischild-2019-317630

**Published:** 2019-08-28

**Authors:** Patrick James Berkeley Walker, Ayobami Adebayo Bakare, Adejumoke Idowu Ayede, Rosena Olubanke Oluwafemi, Omolayo Adebukola Olubosede, Iyabo Victoria Olafimihan, Kenneth Tan, Trevor Duke, Adegoke Gbadegesin Falade, Hamish Graham

**Affiliations:** 1 Centre for International Child Health, Department of Paediatrics, The University of Melbourne, Parkville, Victoria, Australia; 2 Medicine, Nursing & Health Sciences, Monash University, Clayton, Victoria, Australia; 3 Department of Paediatrics, University of Ibadan, Ibadan, Nigeria; 4 University College Hospital Ibadan, Ibadan, Nigeria; 5 Department of Neonatology, Mother and Child Hospital, Akure, Nigeria; 6 Teaching Hospital Complex, University of Medical Sciences, Akure, Nigeria; 7 Department of Paediatrics, Sacred Heart Hospital, Abeokuta, Nigeria; 8 Department of Paediatrics, Monash University, Melbourne, Victoria, Australia; 9 Monash Newborn, Monash Children's Hospital, Melbourne, Victoria, Australia; 10 Intensive Care Unit and University of Melbourne Department of Paediatrics, Royal Children's Hospital, Parkville, Victoria, Australia; 11 Department of Child Health, School of Medicine and Health Sciences, University of Papua New Guinea, Port Moresby, Papua New Guinea; 12 College of Medicine, University of Ibadan, Ibadan, Nigeria; 13 Department of Paediatrics, University College Hospital Ibadan, Ibadan, Nigeria; 14 Centre for International Child Health, University of Melbourne, Royal Children's Hospital, Parkville, Victoria, Australia

**Keywords:** oxygen, oximetry, neonatology, SpO_2_, low-resource

## Abstract

**Objective:**

To evaluate the effectiveness of intermittent pulse oximetry in guiding oxygen therapy in neonates in a low-resource setting.

**Design and setting:**

Prospective validation study at three hospitals in southwest Nigeria. We performed concealed continuous pulse oximetry on participants to evaluate intermittent SpO_2_ monitoring.

**Patients:**

We recruited all preterm or low birthweight neonates, and all term neonates who required oxygen therapy, who were admitted to the neonatal ward(s) of the study hospitals during the study period.

**Main outcome measures:**

Proportion of time preterm/low birthweight neonates on oxygen spent within, above and below the target SpO_2_ range of 90%–95%; and the proportion of time term neonates and neonates not on oxygen spent within and below the target range of 90%–100%.

**Results:**

Preterm/low birthweight neonates receiving oxygen therapy (group A) spent 15.7% (95% CI 13.3 to 18.9) of time in the target SpO_2_ range of 90%–95%. They spent 75.0% (63.6–81.1) of time above 95%, and 2.7% (1.7–5.6) of time below 85%. Term neonates and all neonates not receiving oxygen (group B) spent 97.3% (95% CI 96.4 to 98.6) of time within the target range of 90%–100%, and 0.9% (0.3–1.4) of time below 85%. Guidelines recommended SpO_2_ monitoring 3 times per day for all patients, however neonates in groups A and B were monitored an average of 4.7 and 5.3 times per day, respectively.

**Conclusions:**

To better maintain SpO_2_ within the target range, preterm/low birthweight neonates on oxygen should have their SpO_2_ monitored more frequently than the current 4.7 times per day. In all other neonates, however, monitoring SpO_2_ 5.3 times per day appears suitable.

**What is already known on this topic?**

- Oxygen is an essential medical therapy, but must be used judiciously in preterm and low birthweight neonates to avoid harm from retinopathy of prematurity (ROP) and bronchopulmonary dysplasia (BPD).
- This requires monitoring via pulse oximetry, to keep SpO_2_ within a target range of 90%–95%.
- In many resource-poor settings, continuous pulse oximetry is not available, necessitating the use of intermittent monitoring to guide oxygen therapy.

**What this study adds?**

- We found that preterm and low birthweight neonates spent only 15.7% of time within the target SpO_2_ range of 90%–95% while receiving oxygen, spending 75% of time above 95%.
- Term neonates, however, spent almost all of their time within the target range.
- In preterm and low birthweight neonates receiving oxygen therapy, more frequent SpO_2_ monitoring could improve oxygen targeting and potentially prevent harm from ROP and BPD in resource-constrained hospitals.

## Kudos summary

This study sought to evaluate the effectiveness of intermittent pulse oximetry in guiding oxygen therapy in newborns in a low-resource setting. We conducted the study in three secondary-level hospitals in southwest Nigeria, where oxygen is available for use in newborns, but continuous monitoring of oxygen saturations is not available.

We found that preterm and low birthweight newborns receiving oxygen therapy, who have a narrow oxygen saturation target range of 90%–95%, only spent 15.7% of time within the target range. They spent 75% of time with saturations above 95%, exposing them to potentially dangerous oxygen levels, which can lead to damage to their eyes and lungs. Term neonates and all neonates who were not receiving oxygen, however, spent almost all of their time (97.3%) within their wider target range of 90%–100%.

These results show that to improve oxygen targeting, preterm and low birthweight newborns who are receiving oxygen should have their oxygen saturations more frequently monitored where this is possible. They also demonstrate a need to teach health workers in resource-constrained hospitals about the dangers of using too much oxygen in these patients, and how to respond to oxygen saturation readings above and below the target range.

## Introduction

Oxygen is a life-saving medical therapy for sick newborns that has been used for over 100 years.^1^ When used in preterm (gestational age <37 weeks) and low birthweight (birth weight <2000 g) neonates, however, it carries significant risks—most notably retinopathy of prematurity (ROP) and bronchopulmonary dysplasia (BPD).^2 3^ Tight control of haemoglobin oxygen saturation (SpO_2_) through use of pulse oximetry can prevent harm.^2 4^

The principal and most studied complication of hyperoxia in preterm neonates is ROP. In high-income countries (HICs), ROP is now uncommon due to effective SpO_2_ control in at-risk neonates.^5^ However, its prevalence is increasing in low-income and middle-income countries (LMICs) as neonatal preterm survival increases and oxygen is used excessively. This has been labelled the ‘third epidemic’ of ROP, and is thought to be responsible for 20 000 new cases of blindness annually.^6 7^

While there continues to be some debate about the optimal SpO_2_ range for preterm and low birthweight neonates, it is thought to lie between approximately 90% and 95%,^2 4^ with WHO recommending 88%–95%.^8^ However, it is challenging to maintain SpO_2_ of preterm and low birthweight infants within these target ranges, and they often spend large proportions of time outside.^9–11^ SpO_2_ targeting is particularly challenging in LMICs. Despite improvements in recent years, oxygen practices in many LMICs remain poor and pulse oximetry is rarely used to guide therapy. Hospital surveys consistently report very low availability of pulse oximeters for paediatric and neonatal use (typically <10%) and low staff awareness of how pulse oximetry should be used in children and newborns.^12–16^

In these settings, continuous SpO_2_ monitoring is often not possible and intermittent monitoring is increasingly used to guide oxygen therapy. However, it is unclear how effective it is in guiding oxygen therapy, particularly in neonates at risk of ROP and BPD.

This study seeks to evaluate the effectiveness of intermittent SpO_2_ monitoring in guiding oxygen therapy in neonates in a low-resource setting.

## Methodology

### Study design

We conducted a prospective validation study at three secondary-level hospitals in southwest Nigeria, between June 2017 and March 2018. We evaluated the effectiveness of intermittent pulse oximetry monitoring (the current standard of care at all study hospitals) in guiding oxygen therapy in neonates by performing concealed continuous pulse oximetry on participants during routine care.

### Participants

We recruited all preterm or low birthweight neonates, and all term neonates who required oxygen therapy, who were admitted to the neonatal ward(s) of the study hospitals during the study period. Inclusion and exclusion criteria are detailed in table 1.

**Table 1:** Inclusion and exclusion criteria for participants at all study hospitals

Inclusion criteria	Exclusion criteria
All of: Admitted to nursery of study hospitals during the study period. Preterm or low birth weight; or received oxygen during admission.	Any of: Prescribed or required bubble CPAP, high-flow oxygen (>2 L/min), or mechanical ventilation. Received palliative care.

. CPAP, continuous positive airway pressure.

### Procedures

Intermittent pulse oximetry using Lifebox pulse oximeters (Acare Technology, Taiwan) was a standard practice in all hospitals. We did not provide additional refresher training or reminders, as we intended to evaluate routine care. Staff had previously received training and supportive supervision on pulse oximetry and the oxygen protocol in November 2015.^16 17^ The oxygen protocol recommended using pulse oximetry for every neonate admitted to the nursery, and commencement of oxygen therapy if any SpO_2_ reading was <90%. Pulse oximeter probes were attached to neonates’ hands or feet and nurses recorded the SpO_2_ reading in the patient’s clinical observation chart after an adequate plethysmographic waveform was observed and the SpO_2_ reading was stable (typically within 1–2 min).^16^ The protocol recommended checking the SpO_2_ on admission, within 15 min of any change in oxygen flow rate, and at least once per shift (3 times per day), or more frequently for neonates with severe respiratory distress or signs of deterioration. It recommended aiming for SpO_2_ 90%–95% for preterm or low birthweight neonates receiving oxygen therapy and SpO_2_ ≥90% for other neonates. Monitoring data had shown consistently high use of pulse oximetry for neonates on admission (>90%) and strong adoption into routine care practices.^16^

Data collection at each site was performed by a dedicated study nurse or PJBW, under the supervision of the project coordinator and co-principal investigators. We commenced concealed continuous SpO_2_ monitoring using Masimo Radical-7 CO-Oximeters (Masimo, Irvine, California, USA) after admission to the neonatal ward (or after commencement of oxygen therapy for term neonates), and continued it until 48 hours after oxygen was ceased, or to a maximum of 5 days (120 hours) for each patient. We concealed the visual display and disabled normal upper and lower limit alarms of the Masimo continuous oximeters so SpO_2_ readings could not be determined by staff or study investigators. However, to prevent avoidable harm from severe hypoxaemia occurring, we set a lower audible SpO_2_ alarm limit at 80%, alerting nurses to potentially dangerously low SpO_2_ and prompting clinical review. We also ensured the plethysmographic waveform was visible to permit us to evaluate the accuracy of SpO_2_ readings. We collected data from the first study hospital (SH1) between 22 June 2017 and 31 July 2017, and the second and third study hospitals (SH2, SH3) between 29 November 2017 and 20 March 2018.

We downloaded SpO_2_ data using Profox oximetry software (Profox Associates, Coral Springs, Florida, USA), yielding.xlsx files, which we opened using Stata V.14.0 (StataCorp, College Station, Texas, USA) to enable cleaning and analysis.

### Outcomes

The primary outcome for this study was the proportion of time preterm/low birthweight neonates spent in the target SpO_2_ range (90%–95%) while receiving oxygen therapy. Secondary outcomes were the proportion of time spent by 1) preterm/low birthweight neonates and 2) term neonates:

1. Below safe levels (SpO_2_ <85%);
2. Within safe range (SpO_2_ 85%–95% for preterm/low birthweight neonates on oxygen; 85%–100% for term neonates, or any neonates not on oxygen);
3. Within target range (SpO_2_ 90%–95% for preterm/low birthweight neonates on oxygen; 90%–100% for term neonates, or any neonates not on oxygen);
4. Above safe levels (SpO_2_ >95% for preterm/low birthweight neonates on oxygen).

### Analysis

We stratified participants into those with a target SpO_2_ range of 90%–95% (preterm/low birthweight neonates on oxygen) and those with a target range of 90%–100% (all other neonates). We then reported the primary and secondary outcomes as median values with 95% CIs, as data were not normally distributed. We used Stata V.14.0 to conduct all data cleaning and analysis.

### Ethical aspects

This study was conducted in accordance with the Australian National Statement on Ethical Conduct in Human Research. We obtained written informed consent from a parent/guardian of all participants before enrolment in this study.

## Results

We recruited a total of 86 eligible neonates for this study: 41 from SH1, 26 from SH2 and 19 from SH3. Fifty-one participants were either preterm or low birth weight (19 at SH1, 17 at SH2, 15 at SH3), and 70 received oxygen during their admission (35 at SH1, 26 at SH2, 9 at SH3). Patient characteristics are detailed in table 2.

**Table 2:** Patient characteristics for all participants at all study hospitals

	Preterm or LBW (n=50)	Term (n=36)
Sex	24 (48%) male	22 (61%) male
Hypoxaemic (SpO_2_ <90%) at any time during admission	35 (70%)	26 (72%)
Received oxygen at any time during admission	34 (68%)	36 (100%)
Respiratory distress or tachypnoea (RR >60) at admission	15 (30%)	14 (39%)
Received antibiotics on day 1 of admission	44 (88%)	30 (83%)
Discharged well	27 (54%)	25 (69%)
Discharged against medical advice	6 (12%)	4 (11%)
Died in hospital	13 (26%)	5 (14%)
**Admission diagnoses (including comorbidities**)		
Preterm	20 (40%) 2 (4%) very preterm (GA 28–32 weeks) 4 (8%) extremely preterm (GA <28 weeks)	N/A
Low birth weight	47 (94%) 19 (37%) VLBW 2 (4%) ELBW	N/A
Apnoea	22 (44%)	13 (36%)
Neonatal encephalopathy	11 (22%)	27 (75%)
Suspected neonatal sepsis	24 (48%)	15 (42%)

BW, birth weight; extremely low birth weight (ELBW), BW <1000 g; GA, gestational age; low birth weight (LBW), BW <2000 g;N/A, not applicable; preterm, GA <37 weeks; RR, respiratory rate; very low birthweight (VLBW), BW 1000-1499g.

We monitored participants’ SpO_2_ for a total of 5552 hours (231.3 patient-days). Of this, 4000 hours (166.7 days, 72.1% of total recording time) were for preterm/low birthweight neonates, including 1766 hours (73.6 days; 31.8%) while receiving oxygen; 1551 hours (64.6 days, 27.9%) were for term neonates.

### Frequency of monitoring

During the study period, nurses recorded 1147 intermittent SpO_2_ readings on participants (mean 5.0 readings per patient per day) during routine care: 717 on preterm/low birthweight neonates (mean: 4.3 readings per patient per day; 4.7 while on oxygen; 4.2 while not on oxygen); 430 oximetry readings on term neonates (mean: 6.7 readings). Patients with a target SpO_2_ range of 90%–95% had their SpO_2_ monitored 4.7 times per day; those with a target range of 90%–100% were monitored 5.3 times per day (both mean values).

### Time within the target range

**Figure 1:**
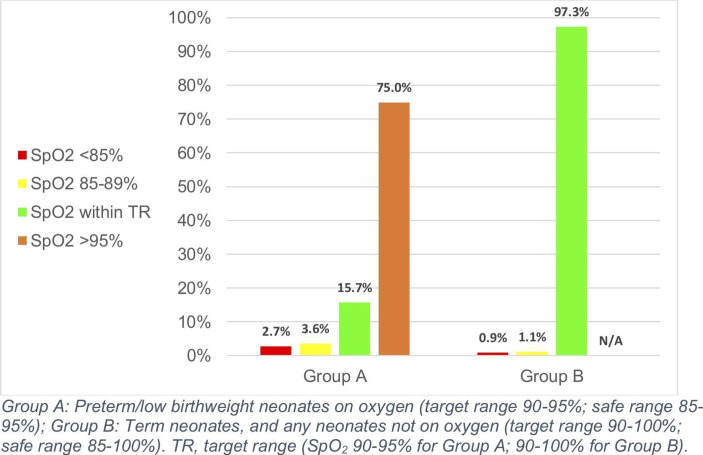
Median proportion of time participants spent within and outside of target SpO_2_ ranges during the study period.

Preterm and low birthweight neonates on oxygen spent only 15.7% (95% CI 13.3 to 18.9) of time within the target SpO_2_ range of 90%–95%. These neonates spent 75.0% of their time (63.6–81.1) with SpO_2_ >95%, and 2.7% (1.7–5.6) of their time with SpO_2_ <85% (table 3, figure 1). Term neonates, and all neonates not on oxygen, however, spent 97.3% (95% CI 96.4 to 98.7) of their time within the target range of 90%–100%, and only 0.9% (0.3–1.4) of their time with SpO_2_ below 85%. Analysing both groups together, participants spent on average 92.5% (95% CI 78.5 to 96.3) of time within the target range, and only 1.5% (1.0–2.3) of time with SpO_2_ <85%.

**Table 3:** Proportion of time participants spent within and outside target and safe SpO_2_ ranges during study period

	Group A (preterm/LBW neonates on oxygen): median (95% CI)	Group B (term neonates, and any neonates not on oxygen): median (95% CI)
Below safe SpO_2_ levels (<85%)	2.7% (1.7 to 5.6)	0.9% (0.3 to 1.4)
	SH1: 3.3 (0.4 to 15.4)	SH1: 0.3 (0.1 to 0.8)
	SH2: 2.4 (1.3 to 8.3)	SH2: 1.8 (0.3 to 24.3)
	SH3: 2.8 (2.5 to 7.6)	SH3: 1.4 (0.9 to 2.9)
Within safe SpO_2_ range (85%–95% or 85%–100%)	19.3% (16.2 to 24.3)	99.1% (98.5 to 99.5)
	SH1: 16.8 (7.8 to 23.3)	SH1: 99.5 (99.1 to 99.7)
	SH2: 24.5 (11.1 to 40.5)	SH2: 98.2 (75.7 to 99.7)
	SH3: 19.2 (10.9 to 24.1)	SH3: 98.6 (97.1 to 99.1)
Within target SpO_2_ range (90%–95% or 90%–100%)	15.7% (13.3 to 18.9)	97.3% (96.4 to 98.7)
	SH1: 15.4 (6.7 to 18.7)	SH1: 98.9 (97.0 to 99.2)
	SH2: 17.0 (10.2 to 24.4)	SH2: 95.8 (62.8 to 98.3)
	SH3: 16.2 (9.5 to 20.5)	SH3: 96.1 (91.7 to 97.4)
Above target SpO_2_ levels (>95%)	75.0% (63.6 to 81.1)	N/A
	SH1: 78.5 (62.3 to 88.9)	
	SH2: 67.1 (40.0 to 81.3)	
	SH3: 78.1 (68.4 to 86.4)	

LBW, low birth weight; N/A, not applicable; SH1/SH2/SH3, study hospitals 1, 2 and 3.

Compared with concealed continuous SpO_2_ monitoring, nurses’ oximetry records detected more time below the target range and less time above the target range (table 4). This was most evident in preterm infants on oxygen therapy.

**Table 4:** Proportion of time participants spent within and outside target and safe ranges, comparing data from concealed continuous SpO_2_ monitoring and nurses’ clinical records from intermittent monitoring

	All patients: median (95% CI)	Group A: median (95% CI)	Group B: median (95% CI)
As per continuous monitoring			
Below safe range	1.5% (1.0 to 2.3)	2.7% (1.7 to 5.6)	0.9% (0.3 to 1.4)
Within safe range	96.2% (83.9 to 98.5)	19.3% (16.2 to 24.3)	99.1% (98.5 to 99.5)
Within target range	92.5% (78.5 to 96.3)	15.7% (13.3 to 18.9)	97.3% (96.4 to 98.7)
Above target range	N/A	75.0% (63.6 to 81.1)	N/A
As per nurses’ record of intermittent monitoring			
Below safe range	7.3%	16.0%	3.6%
Within safe range	79.3%	45.8%	96.4%
Within target range	73.1%	33.2%	92.8%
Above target range	N/A	38.2%	N/A

Group A: preterm/low birthweight neonates on oxygen (target range 90%–95%; safe range 85%–95%). Group B: term neonates, and any neonates not on oxygen (target range 90%–100%; safe range 85%–100%).

N/A, not applicable.

## Discussion

Our findings suggest that current oximetry and oxygen practices in the study hospitals are appropriately maintaining SpO_2_ in the target range for term neonates, but are not adequately restricting oxygen therapy for preterm and low birthweight neonates. While term neonates spent 97% of time in the target range (90%–100%), preterm/low birthweight neonates spent 15.7% of time in the target range (90%–95%) while receiving oxygen. Neonates spent very little (<2%) time below safe levels (<85%), suggesting that oxygen therapy is being used effectively to prevent and treat hypoxia and its potentially lethal sequelae.^18^ However, preterm neonates on oxygen therapy spent 75% of time above the target range (>95%). We did not measure ROP or BPD in our study, but the link between iatrogenic hyperoxia and these conditions is well-established^4 19–21^ and excessive oxygen administration in the study hospitals therefore likely put these neonates at risk of harm.

Nurses’ intermittent SpO_2_ recordings tended to overestimate the time spent below the target range and underestimate the time spent above the target range. This may indicate that nurses more actively look for (and act on) hypoxia than hyperoxia—a hypothesis supported by feedback from nurses at SH1 and previous studies.^22 23^ This has implications for auditing and designing oxygen systems in low-resource hospitals, where relying on recorded SpO_2_ readings may overestimate the time spent under target range, and underestimate the time spent above.

The Lifebox pulse oximeters used by nurses in this study were developed for low-resource settings by WHO and the World Federation of Societies for Anaesthesiologists, with rated accuracy of ±2%.^24 25^ They have been validated for use in children and neonates, and give comparable readings with leading commercial oximetry brands.^24 26^

Existing evidence shows that even in resource-rich settings, it is difficult to control SpO_2_ accurately in preterm and low birthweight neonates. Data from large oxygen-targeting trials among extremely preterm neonates in HICs found that neonates spent 41%–67% of time outside the target range.^9–11 27 28^ Oxygen targeting in our study was expectedly poorer than these studies, which were conducted in well-resourced settings using continuous pulse oximetry monitoring by dedicated neonatal intensive care nurses. Data from Kenya involving intermittent SpO_2_ monitoring found that in the first 24 hours of life, only 6.7% of preterm/low birthweight neonates remained consistently within the target SpO_2_ range, and more than half (53%) had more SpO_2_ readings outside the target range than within.^29^

Common oxygen-related and oximetry-related challenges facing clinicians caring for neonates in resource-poor environments include:

1. Decreased capacity to detect hypoxic or hyperoxic episodes, which can increase the risk of ROP^30^;
2. Nurse:patient ratios as low as 1–2 nurses to 40–50 neonates^27^;
3. Suboptimal staff education in oxygen administration to preterm/low birthweight neonates;
4. Tendency to favour hyperoxia over hypoxia. This observation is consistent with previous studies, and is thought to be related to the innocuous clinical presentation of hyperoxia compared with hypoxia.^9 11 27 28^

### Improving oximetry and oxygen practices in a low-resource environment

Improved access to oxygen therapy globally has been associated with decreased neonatal mortality in many LMICs (particularly MICs).^31^ However, it has also fuelled a rise in the prevalence of ROP.^6 32 33^ Many hospitals now face challenges in the safe use of oxygen in preterm/low birthweight neonates. Our study shows that while pulse oximetry is an essential tool for promoting safe oxygen use, additional measures may need to be introduced for preterm and low birthweight neonates, who are most at risk of harm.

Potential avenues for improvement relate to the frequency of SpO_2_ monitoring, and adequate resource-provision and staffing of neonatal wards. The most financially feasible of these is likely frequency of SpO_2_ monitoring. As this study shows, checking SpO_2_ of neonates with a target range of 90%–100% 5.3 times per day is sufficient to keep SpO_2_ within the desired range. However, in preterm/low birthweight neonates receiving oxygen, monitoring SpO_2_ more frequently than the current 4.7 times per day could lead to improved SpO_2_ control. Informal feedback from nurses at SH1 suggested that monitoring these neonates’ SpO_2_ up to 12 times per day could be feasible, particularly as this change would only involve a minority of neonates. Structural changes such as designated high-dependency zones in neonatal wards with greater nursing resources could help achieve this. All changes to practice should, however, be determined by what is feasible and appropriate in each individual clinical setting.

Recently, automated closed-loop systems have been shown in high-income settings to enable superior SpO_2_ control in preterm/low birthweight neonates.^34–36^ Further investigation is needed to ensure quality and safety of such systems, but application of this technology to simple oxygen systems in resource-constrained settings remains an exciting possibility.

Finally, many commonly used oxygen and oximetry guidelines, including those by WHO, do not make specific reference to how intermittent pulse oximetry should be used in neonates.^8^ Given the increasing use of this monitoring method in LMICs, there is future potential to include clearer guidance on its optimal use in neonates, particularly those born preterm or with low birth weight.

### Limitations

This study has several limitations. First, the number of neonates enrolled in this study was low and involved only three hospitals. Furthermore, few participants were very/extremely preterm or very low/extremely low birth weight, the demographic most at risk of hyperoxic injury. Additional data in this patient group from low-income settings would be helpful to guide future oxygen practices. The study hospitals were also participating in an oxygen improvement project, and had already improved their oxygen practices substantially prior to this study. In the 2 years prior to this study, all three hospitals had adopted pulse oximetry into routine practice, and installed oxygen delivery systems that enabled them to more easily and accurately provide oxygen to patients (including 0-2 LPM flowmeters, nasal prongs, oxygen concentrators and reliable power supply).^16 17^ Unpublished data show that the introduction of pulse oximetry did improve the quality of oxygen care in our study hospitals (personal correspondence, Dr Hamish Graham, January 2019). As such, these hospitals represent relatively good oxygen and pulse oximetry practices compared with most similarly resourced hospitals.

## Conclusions

In this study, we found that in preterm and low birthweight neonates receiving oxygen therapy, monitoring SpO_2_ intermittently an average of 4.7 times per day using a simple decision-making algorithm was only sufficient to keep SpO_2_ within the target range of 90%–95% for a small proportion of time. However, the same procedure enabled nurses to adequately maintain all other neonates’ SpO_2_ within a wider target range of 90%–100%. Preterm and low birthweight neonates on oxygen therapy spent 75% of time in hyperoxia, indicating a need to teach health workers to be alert to the dangers of hyperoxia and the safe upper threshold in these infants, and to enable them to monitor SpO_2_ frequently. Either intermittent or continuous pulse oximetry must be associated with adequate awareness and responses to avoid dangerous hypoxaemia or hyperoxia.
